# Effects of Common Mycorrhizal Network on Plant Carbohydrates and Soil Properties in Trifoliate Orange–White Clover Association

**DOI:** 10.1371/journal.pone.0142371

**Published:** 2015-11-10

**Authors:** Ze-Zhi Zhang, You-Gen Lou, Dao-Juan Deng, Mohammed Mahabubur Rahman, Qiang-Sheng Wu

**Affiliations:** 1 College of Horticulture and Gardening, Yangtze University, Jingzhou, Hubei, People’s Republic of China; 2 Institute of Root Biology, Yangtze University, Jingzhou, Hubei, People’s Republic of China; 3 School of Foreign Studies, Yangtze University, Jingzhou, Hubei, People’s Republic of China; 4 Brix N’ Berries, Leduc, Alberta, Canada; South China Agricultural University, CHINA

## Abstract

Common mycorrhizal network (CMN) allows nutrients and signals to pass between two or more plants. In this study, trifoliate orange (*Poncirus trifoliata*) and white clover (*Trifolium repens*) were planted in a two-compartmented rootbox, separated by a 37–μm nylon mesh and then inoculated with an arbuscular mycorrhizal fungus (AMF), *Diversispora spurca*. Inoculation with *D*. *spurca* resulted in formation of a CMN between trifoliate orange and white clover, whilst the best AM colonization occurred in the donor trifoliate orange–receptor white clover association. In the trifoliate orange–white clover association, the mycorrhizal colonization of receptor plant by extraradical hyphae originated from the donor plant significantly increased shoot and root fresh weight and chlorophyll concentration of the receptor plant. Enzymatic activity of soil β-glucoside hydrolase, protease, acid and neutral phosphatase, water-stable aggregate percentage at 2–4 and 0.5–1 mm size, and mean weight diameter in the rhizosphere of the receptor plant also increased. The hyphae of CMN released more easily-extractable glomalin-related soil protein and total glomalin-related soil protein into the receptor rhizosphere, which represented a significantly positive correlation with aggregate stability. AMF inoculation exhibited diverse changes in leaf and root sucrose concentration in the donor plant, and AM colonization by CMN conferred a significant increase of root glucose in the receptor plant. These results suggested that CMN formed in the trifoliate orange–white clover association, and root AM colonization by CMN promoted plant growth, root glucose accumulation, and rhizospheric soil properties in the receptor plant.

## Introduction

Arbuscular mycorrhiza (AM) is a mutual symbiosis formed between the roots of ~80% of terrestrial plants and soil arbuscular mycorrhizal fungi (AMF). This symbiosis can assist the host plant to absorb water and nutrients from the soil. AMs produce well-developed extraradical mycorrhizal mycelium that can connect the roots of either different plant species or the same species, leading to the formation of a common mycorrhizal network (CMN) between their neighbors [1, 2]. A CMN derives from mycorrhizal hyphae colonizing neighboring roots, but also from hyphal anastomoses uniting previously separated mycelia [3]. The hyphal connection between plants would provide pathways for the transfer of nutrients and chemical and electrical signals from one plant to another [4–8]. Application of ^32^N isotope tracer indicated that a CMN between grasses (*Bromus hordeaceus*, *B*. *madritensis* and *Nassella pulchra*) and forbs (*Madia gracilis*, *Sanicula bipinnata*, and *Trifolium microcephalum*) was established by collective AMF, and the CMN represented the N communication between the different plants [4]. Barto et al. [5] revealed the transfer of allelochemicals from supplier to target *Tagetes tenuifolia* plants by CMNs. Therefore, studies of CMNs will reveal communication of the underground assimilation products, nutrients and signal substances for resource sharing between plants.

Among nutrient elements, carbon (C) is transferred between two or more plants through CMNs, resulting in the changes in the physiological activities between plants [9–11]. In general, AMF obtain the photosynthetic carbohydrates of the host plant to carry out its life cycle. In the *Festuca arundinacea* plants inoculated with *Glomus* sp., 41% of ^13^C label transferred from leaf to root in the donor plant and further to the adjacent plants [9]. Another study indicated that ^13^C successfully transferred from the donor plant, *Rhizophagus intraradices*-colonized *Trifolium subterraneum*, to the receptor *T*. *subterraneum* plant, but not the receptor *Plantago lanceolata* plant [10]. Interestingly, a C_3_ plant, flax, invested little C, but would obtain up to 94% of N and P by CMN from a C_4_ plant, sorghum [11]. It indicated that the C allocation by CMN is strongly dependent on the donor plant species.

Soil water-stable aggregate (WSA) distribution is usually considered in the evaluation of soil structure, because WSA can affect soil water and nutrient status and the absorbed roles of plant roots from soil [12]. Extraradical mycelium of arbuscular mycorrhizas releases a special glycoprotein into soil, called glomalin-related soil protein (GRSP) [13]. The GRSP showed an important functioning on cementing soil aggregation and stabilizing soil aggregates [14]. However, no information is available regarding the CMN effect on GRSP production and its function on aggregate stability in rhizosphere of donor and receptor plants.

Trifoliate orange [*Poncirus trifoliata* (L.) Raf.], one of the most important citrus rootstocks used throughout the world, is strongly dependent on AMs, due to a lack or low number of root hairs. In the field, sod culture is widely used in citrus orchards to enhance organic matter and mineral contents in soil, benefit propagation of AMF, and reduce the consumption of agrichemicals and chemical fertilizers [15]. Meanwhile, white clover (*Trifolium repens* L.) as less mycorrhizal dependent plant is widely applied into citrus orchards [16], where it co-exists with trifoliate orange. The information about the CMN establishment and functioning in trifoliate orange–white clover association is not clearly known. The aims of the present study are to assess whether a CMN is formed between trifoliate orange and white clover and to clarify how CMN affects plant biomass, chlorophyll and carbohydrate concentrations, and soil properties in receptor plant.

## Materials and Methods

### Two-compartmented rootbox

A two-compartmented rootbox was made of plexiglass. Each compartment was 6-cm length, 6-cm width, and 17-cm height. A pipe with 2×4×4 cm (length×width×height) size was fixed to a 2-cm location of the compartment bottom. A 37-μm nylon mesh was directly wrapped on the front of the pipe. The two compartments were connected by the two joint pipes and separated from the 37-μm nylon mesh, where AM hyphae, but not plant roots, are allowed from a compartment to the other for the establishment of a CMN.

### Biological materials

The soil used was Ferralsol (FAO system) collected from a citrus orchard of Yangtze University campus. The soil had a pH of 6.0 and 15.7 mg/kg Bray-P. A nine-leaf-old trifoliate orange seedling grown in autoclaved (121°C, 0.11 Mpa, 2 h) sand without AMF was transplanted into a compartment, and then 15 seeds of white clover sterilized with 70% alcohol for 10 min were sown into the other compartment. Approx. 690 g of soil sterilized with 121°C and 0.11 Mpa for 2 h, was supplied into each chamber. The white clover was thinned to 8 plants per chamber after one week of sowing. Approx. 1,050 spores of an AM fungus, *Diversispora spurca* (CM Pfeiff, C Walker & Bloss) C Walker & A Schüßler, were inoculated into the designed compartment at the time of transplanting. The details of the AM fungal strain in origin and propagation were described previously by Wu et al. [17].

All the rootboxes were placed in a glass greenhouse in Yangtze University campus from March 28, 2014 to August 16, 2014, where photosynthetic photon flux density was 740–960 μmol/m^2^/s, day/night temperature 28/21°C, and relative humidity 85%. The position of rootboxes in the greenhouse was rotated weekly. During the experiment, no additional nutrition was added into each chamber of the rootbox, in order to maintain a considerably low soil P level for better AM functioning.

### Experimental design

The experiment consisted of four treatments with a completely randomized blocked arrangement: (1) both trifoliate orange and white clover plants were inoculated with *D*. *spurca* (TO^+^/WC^+^); (2) the donor plant trifoliate orange was inoculated with *D*. *spurca*, but the receptor plant white clover was not inoculated with *D*. *spurca* (TO^+^/WC^–^); (3) the donor plant white clover was inoculated with *D*. *spurca*, but the receptor plant trifoliate orange was not inoculated with *D*. *spurca* (TO^–^/WC^+^); (4) both trifoliate orange and white clover were not inoculated with *D*. *spurca* (TO^–^/WC^–^). Each treatment had four replicates, creating a total of 16 rootboxes.

### Variable determinations

After 20 weeks of the AMF inoculation, the trifoliate orange and white clover seedlings were harvested and divided into the shoot and the root, whose fresh biomass was determined. The soil adhered to the root systems was collected, air-dried, and sieved through a 4 mm mesh.

Leaf chlorophyll concentration was determined by Lichtenthaler and Wellburn [18] with the extraction of 80% acetone solutions.

Extraction of fructose, glucose, and sucrose in leaves and roots was done with both 8 mL of 80% ethanol and 50 mg of 1.0-mm-sieved dry samples at 80°C for 40 min, which was centrifuged at 2,500×g for 5 min. Concentrations of these carbohydrates were determined as per the colorimetrical protocol described by Wu et al. [19].

Soil β-glucoside hydrolases, proteases, and phosphatase activities were determined respectively by the method of Stot et al. [20], Cao et al. [21], and Zhao et al. [22]. Distribution of water-stable aggregate (WSA) at the size of 0.25–0.5, 0.5–1, 1–2 and 2–4 mm was determined by wet–sieved method [23]. Mean weight diameter (MWD) of WSAs in 0.25–4.00 mm size is an indicator of aggregate stability and calculated as follows [23]:

$$\text{MWD}=\sum_{i=1}^{n}XiWi$$

where, *xi* is the diameter of the *i* sieve (mm), *wi* is the proportion of the *i* size fraction in the total sample mass, and *n* is the number of size fractions (*n* = 4).

The easily-extractable glomalin-related soil protein (EE-GRSP), difficultly-extractable GRSP (DE-GRSP) and total GRSP concentration (T-GRSP) concentration was determined by the protocol of Wu et al. [14].

### Statistical analysis

Data were statistically analyzed with one-factor of variance (ANOVA) in SAS (v 8.1), and the Duncan’s multiple range test at *P*<0.05 was used to compare the significant differences between the treatments.

## Results

### Mycorrhizal colonization and CMN

**Fig 1:**
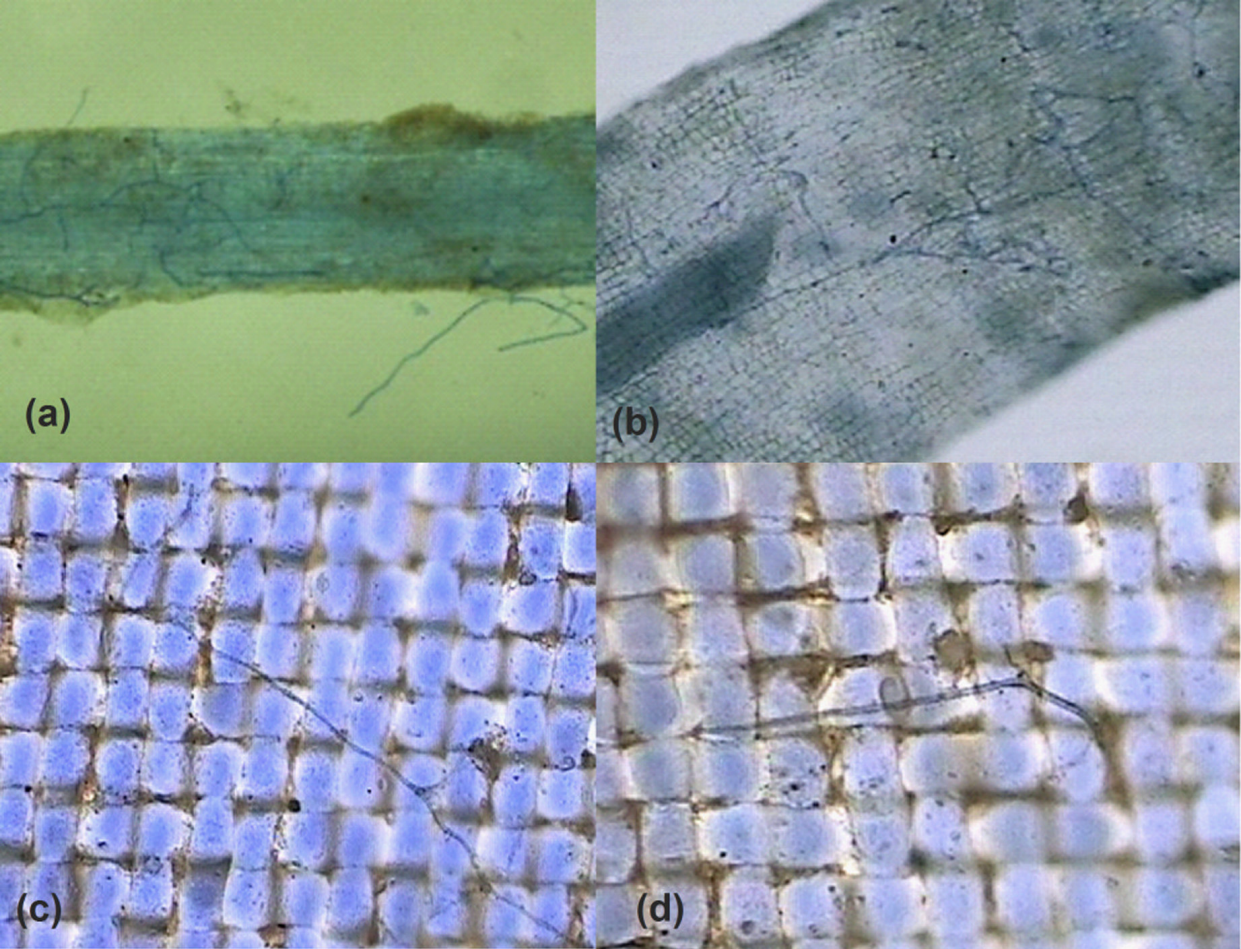
Root AM colonization of white clover (a) and trifoliate orange (b) and the AM hyphae (c and d) in 37 μm nylon-mesh. Meanwhile, a and b subfigure indicated that *Diversispora spurca* entered the root cell to form intraradical hyphae or extraradical hyphae, and c and d subfigure indicated that extraradical mycorrhizal mycelium went through 37-μm nylon-mesh from one compartment to another compartment.

Trifoliate orange and white clover plants were well colonized by *D*. *spurca* (Fig 1A and 1B), and root mycorrhizal colonization was 27.3±0.6, 35.2±5.5, and 23.6±4.9% in trifoliate orange and 27.0±2.8, 58.1±7.5, and 27.1±3.1% in white clover under the TO^+^/WC^+^, TO^+^/WC^–^, and TO^–^/WC^+^ treatments conditions, respectively. A CMN was established between the donor and receptor plants under TO^+^/WC^+^, TO^+^/WC^–^, and TO^–^/WC^+^ conditions, as indicated by the presence of AM extraradical hyphae penetration in 37-μm nylon mesh between the two compartments (Fig 1C and 1D).

### Plant biomass and chlorophyll concentration

As shown in Table 1, inoculation of donor plants with *D*. *spurca* resulted in significant increases in chlorophyll content, shoot fresh weight, and root fresh weight of both donor and receptor plants, compared to non-AMF controls. There was no significant difference in shoot and root fresh weight of white clover and in chlorophyll concentration of trifoliate orange and white clover between the TO^+^/WC^+^, TO^+^/WC^–^, and TO^–^/WC^+^ treatments. However, in trifoliate orange, significantly higher shoot and root fresh weight was ranked as TO^+^/WC^+^ ≥ TO^+^/WC^−^> TO^–^/WC^+^.

**Table 1:** Effects of *Diversispora spurca* inoculation on plant biomass and chlorophyll concentration in trifoliate orange–white clover association in a two-compartmented rootbox separated by 37 μm nylon-mesh.

Treatment	Shoot fresh weight (g FW /plant)		Root fresh weight (g FW/plant)		Chlorophyll concentration (mg/g FW)	
	Trifoliate orange	White clover	Trifoliate orange	White clover	Trifoliate orange	White clover
TO^+^/WC^+^	2.58±0.24a	7.97±0.51a	1.14±0.08a	0.59±0.02a	2.10±0.14a	2.56±0.14a
TO^+^/WC^–^	2.43±0.15a	8.03±0.50a	1.04±0.06b	0.57±0.03a	2.16±0.13a	2.48±0.16a
TO^–^/WC^+^	2.13±0.11b	8.57±0.46a	0.92±0.02c	0.60±0.04a	2.07±0.09a	2.35±0.23a
TO^–^/WC^–^	1.71±0.03c	7.08±0.41b	0.82±0.05d	0.41±0.02b	1.87±0.11b	2.08±0.06b

Data (means ± SD, *n* = 4) followed by different letters indicate significant differences (*P* < 0.05) between treatments. Abbreviation: TO^+^/WC^+^, both trifoliate orange and white clover plants were inoculated with *Diversispora spurca*; TO^+^/WC^–^, the donor plant trifoliate orange was inoculated with *D*. *spurca*, but the receptor plant white clover was not inoculated with *D*. *spurca*; TO^–^/WC^+^, the donor plant white clover was inoculated with *D*. *spurca*, but the receptor plant trifoliate orange was not inoculated with *D*. *spurca*; TO^–^/WC^–^, both trifoliate orange and white clover were not inoculated with *D*. *spurca*.

### Carbohydrate concentration in leaves and roots

**Fig 2:**
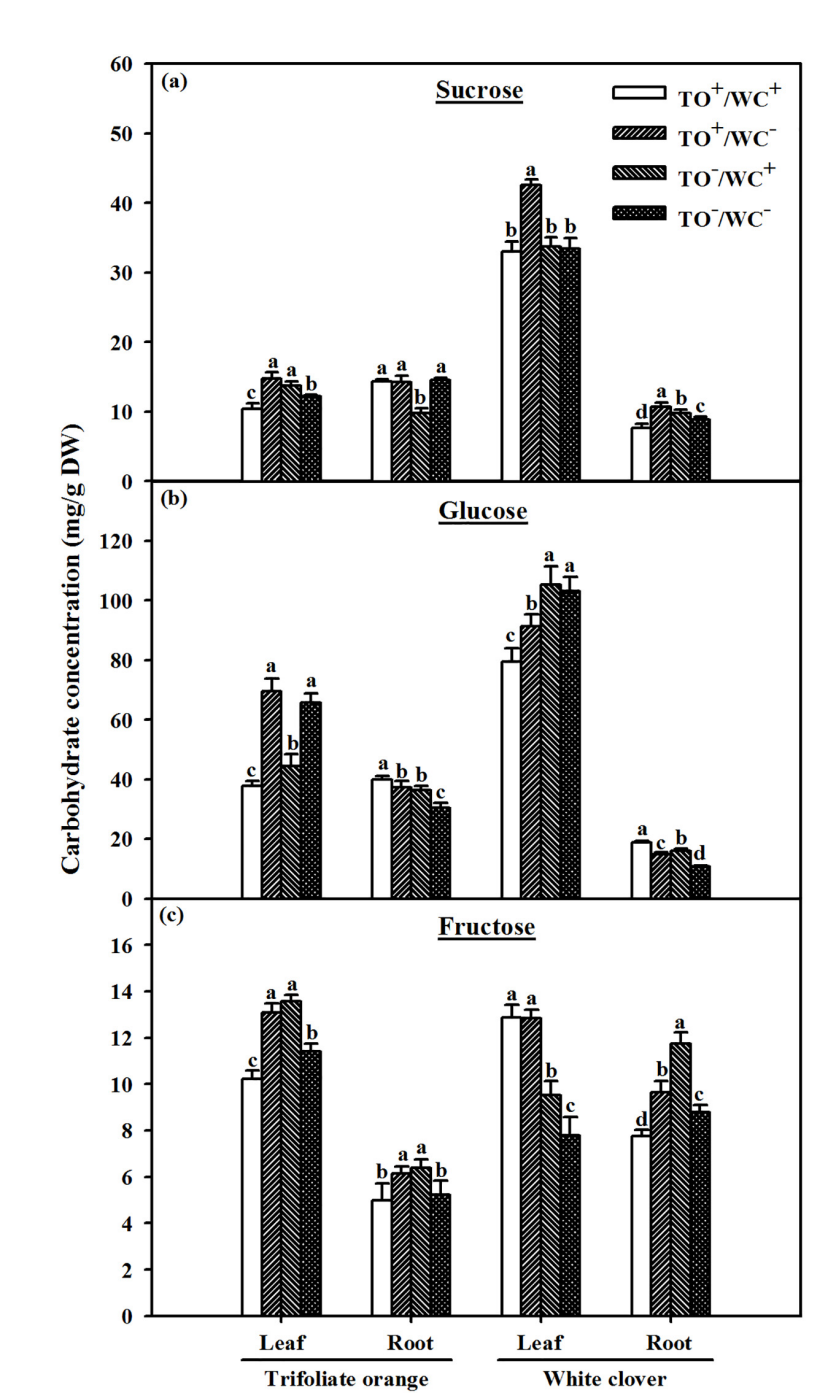
Sucrose (a), glucose (b), and fructose (c) concentrations in leaf and root of trifoliate orange and white clover plants inoculated with *Diversispora spurca* in a two-compartmented rootbox separated by 37 μm nylon-mesh. Data (means ± SD, *n* = 4) followed by different letters indicate significant differences (*P* < 0.05) between treatments. Abbreviation: TO^+^/WC^+^, both trifoliate orange and white clover plants were inoculated with *D*. *spurca*; TO^+^/WC^–^, the donor plant trifoliate orange was inoculated with *D*. *spurca*, but the receptor plant white clover was not inoculated with *D*. *spurca*; TO^–^/WC^+^, the donor plant white clover was inoculated with *D*. *spurca*, but the receptor plant trifoliate orange was not inoculated with *D*. *spurca*; TO^–^/WC^–^, both trifoliate orange and white clover were not inoculated with *D*. *spurca*.

As shown in Fig 2A, significantly higher sucrose concentration in trifoliate orange seedlings with four treatments was rated as TO^+^/WC^–^ ≥ TO^–^/WC^+^ > TO^–^/WC^−^> TO^+^/WC^+^ in leaves and TO^+^/WC^+^ ≈ TO^+^/WC^–^ ≈ TO^–^/WC^–^ ≈ TO^–^/WC^+^ in roots, respectively. In white clover, the change tendency of sucrose concentration among the treatments was TO^+^/WC^−^> TO^+^/WC^+^ ≈ TO^–^/WC^+^ ≈ TO^–^/WC^−^in leaves and TO^+^/WC^−^> TO^–^/WC^+^ > TO^–^/WC^−^> TO^+^/WC^+^ in roots, respectively.

Significant higher glucose and fructose concentrations in leaves of trifoliate orange seedlings among the treatments was TO^+^/WC^–^ ≈ TO^–^/WC^−^> TO^–^/WC^+^ > TO^+^/WC^+^ and TO^+^/WC^–^ ≈ TO^–^/WC^+^ > TO^–^/WC^−^> TO^+^/WC^+^, respectively (Fig 2B and 2C). In white clover, leaf glucose and fructose concentrations with four treatments represented the significant tendency of TO^–^/WC+ ≈ TO^–^/WC^−^> TO^+^/WC^−^> TO^+^/WC^+^ and TO^+^/WC^+^ ≈ TO^+^/WC^−^> TO^–^/WC^+^ > TO^–^/WC^–^. In addition, root glucose and fructose of trifoliate orange among the four treatments ranked as TO^+^/WC^+^ > TO^+^/WC^–^ ≈ TO^–^/WC^+^ > TO^–^/WC^−^and TO^+^/WC^–^ ≈ TO^–^/WC^+^ > TO^–^/WC^–^ ≈ TO^+^/WC^+^, respectively. Significantly higher root glucose and fructose concentrations in white clover ranked as TO^+^/WC^+^ > TO^–^/WC^+^ > TO^+^/WC^−^> TO^−^/WC^−^and TO^–^/WC^+^ > TO^+^/WC^−^> TO^–^/WC^−^> TO^+^/WC^+^, respectively.

### Soil relevant enzyme activities

Significantly higher soil β-glucosidase activity among the four treatments was the trend of TO^–^/WC^+^ ≥ TO^+^/WC^–^ ≥ TO^–^/WC^−^> TO^+^/WC^+^ in rhizosphere of trifoliate orange and TO^+^/WC^–^ ≈ TO^–^/WC^+^ > TO^–^/WC^−^> TO^+^/WC^+^ in rhizosphere of white clover (Table 2).

**Table 2:** Effects of *Diversispora spurca* inoculation on rhizospheric soil β-glucosidase and protease activities in trifoliate orange–white clover association in a two-compartmented rootbox separated by 37 μm nylon-mesh

Treatment	β-glucosidase (mg reduced sugar/g DW soil)		Protease (μg Glycine/g DW soil)	
	Trifoliate orange	White clover	Trifoliate orange	White clover
TO^+^/WC^+^	0.64±0.13c	0.65±0.14c	6.60±0.29b	5.97±0.24b
TO^+^/WC^–^	1.19±0.11ab	1.09±0.10a	6.28±0.36b	5.94±0.23b
TO^–^/WC^+^	1.28±0.13a	1.05±0.03a	7.16±0.40a	6.36±0.30a
TO^–^/WC^–^	1.02±0.14b	0.85±0.07b	4.95±0.26c	5.45±0.16c

Data (means ± SD, *n* = 4) followed by different letters indicate significant differences (*P* < 0.05) between treatments. Abbreviation: TO^+^/WC^+^, both trifoliate orange and white clover plants were inoculated with *Diversispora spurca*; TO^+^/WC^–^, the donor plant trifoliate orange was inoculated with *D*. *spurca*, but the receptor plant white clover was not inoculated with *D*. *spurca*; TO^–^/WC^+^, the donor plant white clover was inoculated with *D*. *spurca*, but the receptor plant trifoliate orange was not inoculated with *D*. *spurca*; TO^–^/WC^–^, both trifoliate orange and white clover were not inoculated with *D*. *spurca*.

The significant variation tendency of soil protease activity on trifoliate orange and white clover among the four treatments ranked as TO^–^/WC^+^ > TO^+^/WC^+^ ≈ TO^+^/WC^−^> TO^–^/WC^−^(Table 2).

Compared with the TO^–^/WC^−^treatment, the TO^+^/WC^+^, TO^+^/WC^−^and TO^–^/WC^+^ treatments significantly increased rhizospheric acid phosphatase and neutral phosphatase activity of trifoliate orange and white clover, whilst the TO^+^/WC^−^treatment represented the greatest effect (Table 3). Soil alkaline phosphatase activity in rhizosphere of trifoliate orange under the TO^+^/WC^+^ was significantly higher than under the other three treatments, while there was no significant difference of alkaline phosphatase activity between the four treatments in white clover rhizosphere.

**Table 3:** Effects of *Diversispora spurca* inoculation on rhizospheric soil acid, neutral and alkaline phosphatase activities (mg hydroxybenzene/g DW soil) in trifoliate orange–white clover association in a two-compartmented rootbox separated by 37 μm nylon-mesh.

Treatment	Acid phosphatase		Neutral phosphatase		Alkaline phosphatase	
	Trifoliate orange	White clover	Trifoliate orange	White clover	Trifoliate orange	White clover
TO^+^/WC^+^	1.66±0.08b	1.34±0.08b	1.70±0.10a	1.25±0.06b	1.81±0.25a	1.40±0.18a
TO^+^/WC^–^	2.08±0.29a	1.64±0.12a	1.64±0.11a	1.55±0.12a	1.27±0.22b	1.38±0.19a
TO^–^/WC^+^	1.37±0.10c	1.45±0.19ab	1.22±0.11b	1.24±0.17b	1.26±0.15b	1.41±0.30a
TO^–^/WC^–^	0.91±0.15d	1.06±0.13c	1.05±0.05c	1.02±0.11c	1.38±0.13b	1.30±0.52a

Data (means ± SD, *n* = 4) followed by different letters indicate significant differences (*P* < 0.05) between treatments. Abbreviation: TO^+^/WC^+^, both trifoliate orange and white clover plants were inoculated with *Diversispora spurca*; TO^+^/WC^–^, the donor plant trifoliate orange was inoculated with *D*. *spurca*, but the receptor plant white clover was not inoculated with *D*. *spurca*; TO^–^/WC^+^, the donor plant white clover was inoculated with *D*. *spurca*, but the receptor plant trifoliate orange was not inoculated with *D*. *spurca*; TO^–^/WC^–^, both trifoliate orange and white clover were not inoculated with *D*. *spurca*.

### Production of GRSP fractions

Compared with the TO^–^/WC^−^treatment, the TO^+^/WC^+^, the TO^+^/WC^–^, and the TO^–^/WC^+^ treatments significantly increased EE-GRSP, DE-GRSP and T-GRSP concentration in rhizosphere of trifoliate orange and white clover plants, except that DE-GRSP in rhizosphere of trifoliate orange was no significant difference among the TO^–^/WC^–^, the TO^+^/WC^–^, and the TO^–^/WC^+^ treatments (Table 4).

**Table 4:** Effects of *Diversispora spurca* inoculation on rhizospheric three glomalin-related soil protein (GRSP, mg/g DW soil) concentrations in trifoliate orange–white clover association in a two-compartmented rootbox separated by 37 μm nylon-mesh.

Treatment	EE-GRSP		DE-GRSP		T-GRSP	
	Trifoliate orange	White clover	Trifoliate orange	White clover	Trifoliate orange	White clover
TO^+^/WC^+^	0.790±0.019a	0.814±0.029a	0.591±0.032a	0.451±0.013a	1.381±0.012a	1.266±0.039a
TO^+^/WC^–^	0.813±0.036a	0.775±0.020b	0.568±0.037ab	0.425±0.008b	1.380±0.048a	1.200±0.023b
TO^–^/WC^+^	0.797±0.020a	0.806±0.008ab	0.578±0.023ab	0.439±0.020ab	1.375±0.034a	1.245±0.014a
TO^–^/WC^–^	0.721±0.022b	0.729±0.019c	0.532±0.024b	0.382±0.013c	1.253±0.040b	1.111±0.019c

Data (means ± SD, *n* = 4) followed by different letters indicate significant differences (*P* < 0.05) between treatments. Abbreviation: TO^+^/WC^+^, both trifoliate orange and white clover plants were inoculated with *Diversispora spurca*; TO^+^/WC^–^, the donor plant trifoliate orange was inoculated with *D*. *spurca*, but the receptor plant white clover was not inoculated with *D*. *spurca*; TO^–^/WC^+^, the donor plant white clover was inoculated with *D*. *spurca*, but the receptor plant trifoliate orange was not inoculated with *D*. *spurca*; TO^–^/WC^–^, both trifoliate orange and white clover were not inoculated with *D*. *spurca*.

### Distribution of WSAs and aggregate stability

**Fig 3:**
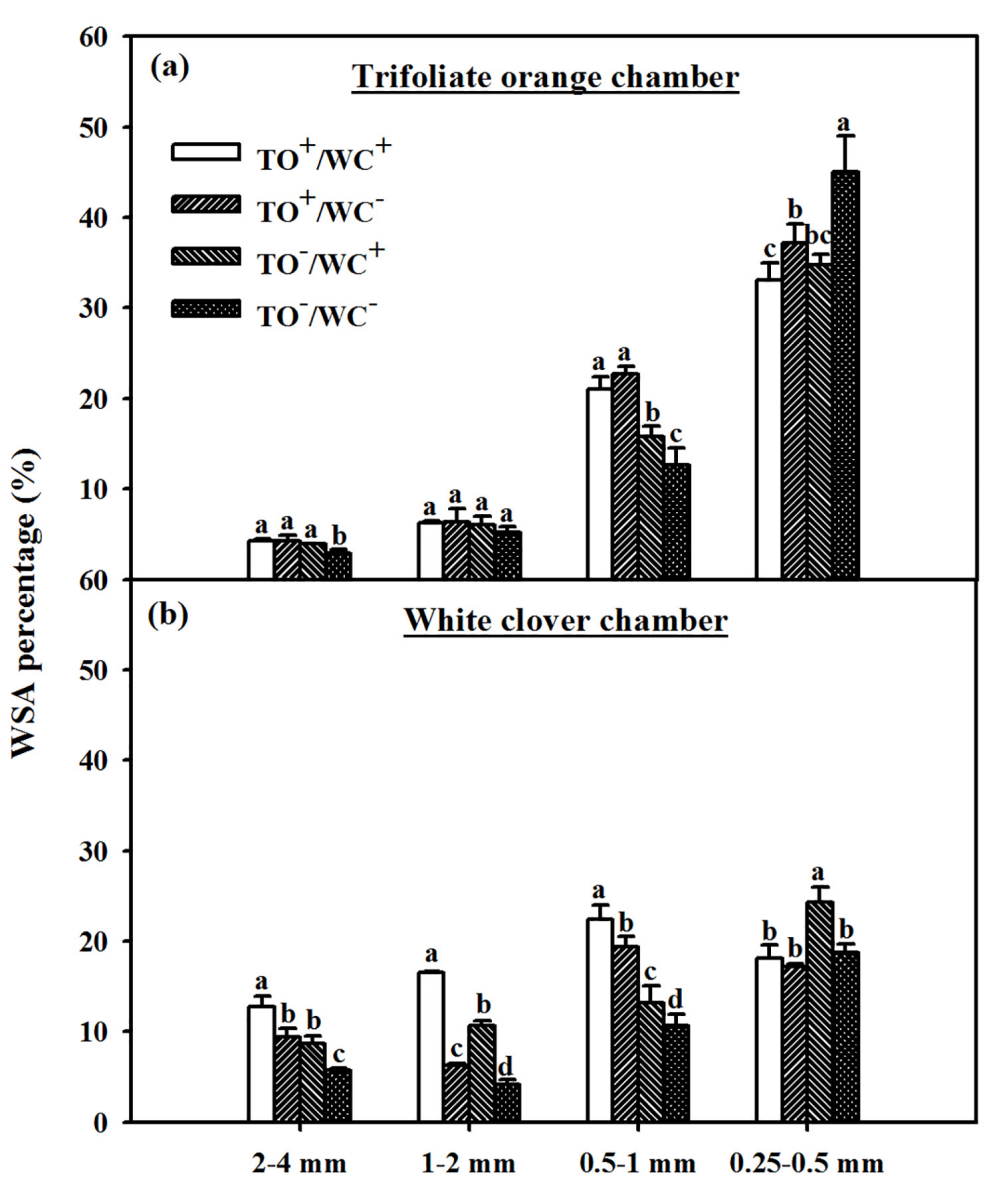
Water-stable aggregate distribution in rhizosphere of trifoliate orange (a) and white clover (b) plants inoculated with *Diversispora spurca* in a two-compartmented rootbox separated by 37 μm nylon-mesh. Data (means ± SD, *n* = 4) followed by different letters indicate significant differences (*P* < 0.05) between treatments. Abbreviation: TO^+^/WC^+^, both trifoliate orange and white clover plants were inoculated with *D*. *spurca*; TO^+^/WC^–^, the donor plant trifoliate orange was inoculated with *D*. *spurca*, but the receptor plant white clover was not inoculated with *D*. *spurca*; TO^–^/WC^+^, the donor plant white clover was inoculated with *D*. *spurca*, but the receptor plant trifoliate orange was not inoculated with *D*. *spurca*; TO^–^/WC^–^, both trifoliate orange and white clover were not inoculated with *D*. *spurca*.

In trifoliate orange compartment, compared with TO^–^/WC^−^treatment, the TO^+^/WC^+^, TO^+^/WC^−^and TO^–^/WC^+^ treatments significantly increased soil WSA percentage at the size of 2–4 and 0.5–1 mm, significantly reduced WSA percentage in 0.25–0.5 mm size, but did not significantly change WSA percentage in 1–2 mm size (Fig 3A).

In white clover compartment, the TO^+^/WC^+^, TO^–^/WC^+^ and TO^+^/WC^−^treatments represented significantly higher WSA percentage at the size of 2–4, 1–2 and 0.5–1 mm than the TO^–^/WC^−^control, whilst the TO^+^/WC^+^ treatment showed the highest effect. Only the TO^–^/WC^+^ treatment significantly increased WSA percentage at 0.25–0.5 mm in white clover rhizosphere than the other three treatments (Fig 3B).

**Fig 4:**
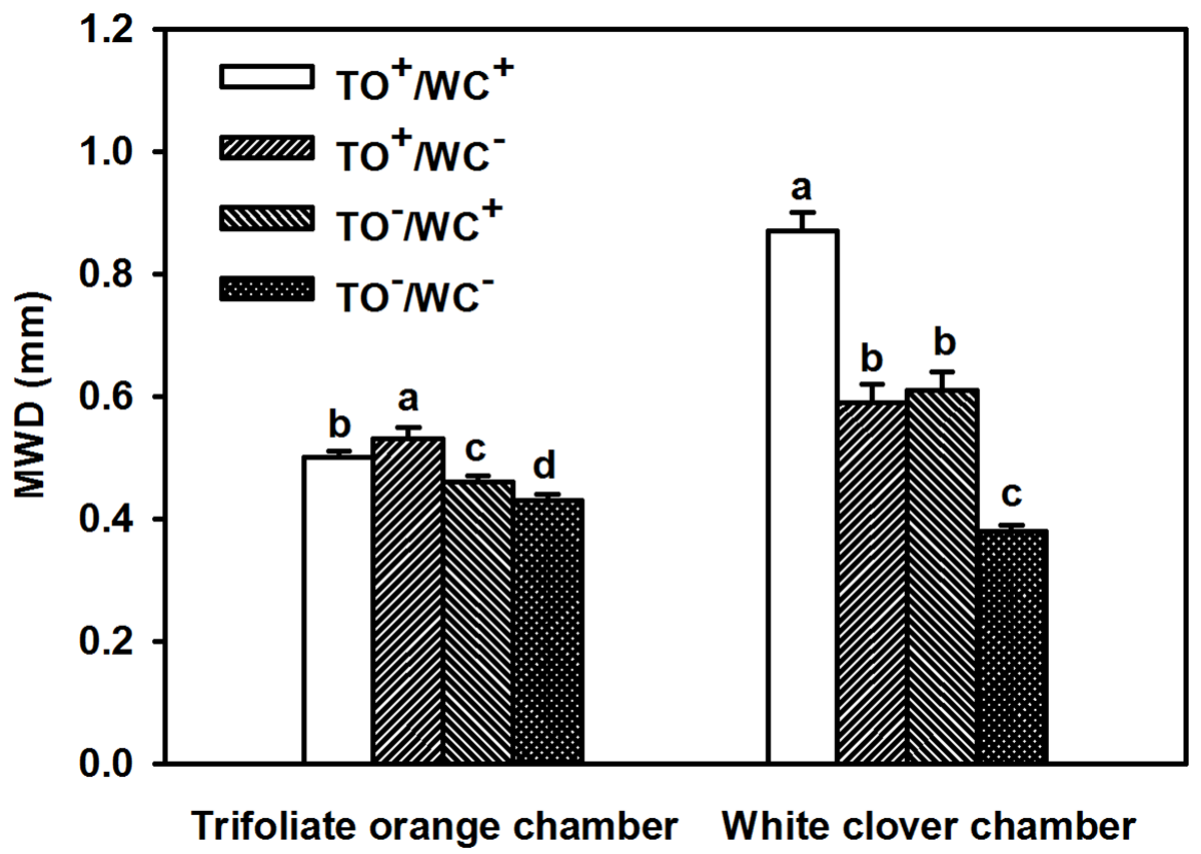
Mean weight diameter (MWD) of water-stable aggregates in 0.25–4 mm size in rhizosphere of trifoliate orange and white clover plants inoculated with *Diversispora spurca* in a two-compartmented rootbox separated by 37 μm nylon-mesh. Data (means ± SD, *n* = 4) followed by different letters indicate significant differences (*P* < 0.05) between treatments. Abbreviation: TO^+^/WC^+^, both trifoliate orange and white clover plants were inoculated with *D*. *spurca*; TO^+^/WC^–^, the donor plant trifoliate orange was inoculated with *D*. *spurca*, but the receptor plant white clover was not inoculated with *D*. *spurca*; TO^–^/WC^+^, the donor plant white clover was inoculated with *D*. *spurca*, but the receptor plant trifoliate orange was not inoculated with *D*. *spurca*; TO^–^/WC^–^, both trifoliate orange and white clover were not inoculated with *D*. *spurca*.

The MWD, an indicator of soil aggregate stability, was significantly higher in rhizosphere of trifoliate orange and white clover under the TO^+^/WC^+^, the TO^+^/WC^–^, and the TO^–^/WC^+^ treatments than under the TO^–^/WC^−^treatment conditions (Fig 4). Meanwhile, the highest MWD was found in rhizosphere of trifoliate orange treated by the TO^+^/WC^−^and white clover treated by the TO^+^/WC^+^.

## Discussion

The present study showed that inoculation with *D*. *spurca* resulted in formation of a CMN between trifoliate orange and white clover, suggesting that CMNs might commonly be found in the sod culture of citrus orchards. Moreover, the AM colonization significantly increased chlorophyll content and shoot and root fresh weight of trifoliate orange and white clover as donor and receptor, which is consistent with the results of Walder et al. [11] in donor flax–receptor sorghum plant association. However, Zhao et al. [24] reported that *Claroideoglomus etunicatum* inoculation did not affect the change in shoot and root fresh weight in watermelon–aerobic rice dual system. Zhu et al. [25] showed that compared to white clover as a donor, inoculation with *F*. *geosporum* in ryegrass as a donor, did not significantly affect the shoot and root biomass of ryegrass and white clover. Meanwhile, a significantly higher plant biomass was found in white clover as a donor inoculated with *F*. *geosporum*, as compared with ryegrass as a donor. It suggests that AMF and the host plant species could strongly affect growth responses in the dual system.

The present work also revealed that the CMN in trifoliate orange–white clover association could significantly increase shoot and root fresh weight of the receptor plant, suggesting that CMN presence would benefit the plant growth of their neighbors, which is related to mineral nutrients (especially phosphorus) transferred through CMN or to the receptor plant infected by CMN. Compared with the TO^–^/WC^+^ treatment, the TO^+^/WC^−^treatment represented significantly higher shoot and root fresh weight in trifoliate orange but considerably lower shoot and root biomass in white clover. One possibility is that trifoliate orange as high mycorrhizal dependent plant is more sensitive to *D*. *spurca* inoculation or colonization by CMN hyphae than white clover. Therefore, the CMN role in fresh biomass production of receptor plant could be strongly dependent on the mycorrhizal dependence of the receptor plant.

In this study, AMF inoculation showed significantly higher leaf sucrose and fructose and root sucrose and glucose in the donor trifoliate orange, and higher leaf sucrose and fructose and higher root sucrose, fructose, and glucose of the receptor white clover. Wu et al. [17, 19] also reported the diverse responses of sucrose, glucose and fructose concentrations to AMF in leaves and roots of trifoliate orange.

In donor white clover–receptor trifoliate orange system, root AM colonization caused by CMN markedly decreased root sucrose but increased glucose and fructose in the receptor plant. Possibly, root colonization caused by CMN in trifoliate orange–white clover association might accelerate root sucrose cleavage into fructose and glucose in the receptor plant, resulting in an increase of root glucose in the receptor plant, which is beneficial to AM colonization and CMN development [19]. A ^14^C or ^13^C labeling study revealed the C transfer by CMN from the donor plant to the receptor plant [9–11], and the C investing represented different return of investment, such N and P acquisition [11]. Further C isotopic labeling studies will be required to highlight the C transfer of a CMN in trifoliate orange–white clover association.

This study indicated that AMF inoculation significantly increased WSA percentage at the size of 2–4 and 0.5–1 mm and soil β-glucoside hydrolases, proteases and acid and neutral phosphatase activities of the donor plant and the receptor plant. Meanwhile, greater soil protease and β-glucoside hydrolases activity in the rhizosphere of donor and receptor plants was under the TO^+^/WC^−^treatment than under the other three treatments. Higher acid and neutral phosphatase activity in the rhizosphere of the donor and receptor plants was under the TO^–^/WC^+^ treatment than under the other three treatments. Greater soil enzyme activities and soil structure by mycorrhization indicated that AMF inoculation conferred better soil structure and soil fertility in mycorrhizosphere. In the present work, significantly higher EE-GRSP and T-GRSP concentration was found in rhizosphere of trifoliate orange–white clover association, irrespective of the donor or receptor plant species. This is in agreement with the findings of Wang et al. [26] in trifoliate orange colonized by different AMF species.

It is well known that mycorrhizal extraradical hyphae could release acid phosphatase [27]. Soil β-glucoside hydrolases, phosphatases and proteases are linked to organic matter, available P and N in soil, respectively [20–22]. In this study, CMN presence significantly increased acid phosphatase, neutral phosphatase, and proteases activities, irrespective of receptor plant species. It suggests that CMN took part in the soil nutrient cycle in receptor rhizosphere. Interestingly, a significantly lower soil β-glucoside hydrolase activity in rhizosphere of trifoliate orange and white clover was found in TO^+^/WC^+^ treatment, as compared with TO^–^/WC^−^treatment. Possibly, under the TO^+^/WC^+^ treatment condition, both trifoliate orange and white clover dispensed more C to maintain own AM development and formed respective CMN to compete with their neighbors in soil C cycle. The present study showed that CMN presence significantly increased EE-GRSP and T-GRSP levels in receptor rhizosphere. This is a known fact that GRSP exhibited a strongly positive correlation with soil aggregate stability under various soil types and ecosystem conditions [13, 14, 28], and soil mycorrhizal hyphae can cement soil aggregate for stabilization [14]. In this study, MWD was significantly positively correlated with EE-GRSP (*r* = 0.58, *P* < 0.05) and T-GRSP (*r* = 0.63, *P* < 0.01) in trifoliate orange chamber and positively with EE-GRSP (*r* = 0.75, *P* < 0.01), DE-GRSP (*r* = 0.82, *P* < 0.01), and T-GRSP (*r* = 0.82, *P* < 0.01) in white clover chamber. It suggests that CMN would induce well-developed mycelium and GRSP release (especially EE-GRSP and T-GRSP) in the receptor plant rhizosphere, resulting in the enhancement of soil aggregate stability, and subsequently promoting the growth of the host plant.

## Conclusion

The CMN with *D*. *spurca* formed in trifoliate orange–white clover association, whilst the best AM colonization occurred in donor trifoliate orange–receptor white clover system. AMF inoculation significantly increased shoot and root fresh biomass, root glucose, EE-GRSP, and T-GRSP concentrations, and soil acid phosphatase, neutral phosphatase, and proteases activities in trifoliate orange or white clover as a donor plant. CMN presence originated from donor plant also holp to enhance plant growth, induce carbohydrate accumulation (mainly glucose in root), and imporve the rhizospheric soil properties in the receiver plant of the trifoliate orange–white clover system. As a result, CMN may exhibit the important functionings in sod culture of citrus orchards.
